# CamPype: an open-source workflow for automated bacterial whole-genome sequencing analysis focused on *Campylobacter*

**DOI:** 10.1186/s12859-023-05414-w

**Published:** 2023-07-20

**Authors:** Irene Ortega-Sanz, José A. Barbero-Aparicio, Antonio Canepa-Oneto, Jordi Rovira, Beatriz Melero

**Affiliations:** 1 Department of Biotechnology and Food Science, University of Burgos, 09006 Burgos, Spain; 2 Department of Computer Science, University of Burgos, 09006 Burgos, Spain

**Keywords:** Pipeline, Comparative genomics, Genome analysis, Bacterial typing, Genome annotation, Virulence genes, Antimicrobial resistance genes

## Abstract

**Background:**

The rapid expansion of Whole-Genome Sequencing has revolutionized the fields of clinical and food microbiology. However, its implementation as a routine laboratory technique remains challenging due to the growth of data at a faster rate than can be effectively analyzed and critical gaps in bioinformatics knowledge.

**Results:**

To address both issues, CamPype was developed as a new bioinformatics workflow for the genomics analysis of sequencing data of bacteria, especially *Campylobacter*, which is the main cause of gastroenteritis worldwide making a negative impact on the economy of the public health systems. CamPype allows fully customization of stages to run and tools to use, including read quality control filtering, read contamination, reads extension and assembly, bacterial typing, genome annotation, searching for antibiotic resistance genes, virulence genes and plasmids, pangenome construction and identification of nucleotide variants. All results are processed and resumed in an interactive HTML report for best data visualization and interpretation.

**Conclusions:**

The minimal user intervention of CamPype makes of this workflow an attractive resource for microbiology laboratories with no expertise in bioinformatics as a first line method for bacterial typing and epidemiological analyses, that would help to reduce the costs of disease outbreaks, or for comparative genomic analyses. CamPype is publicly available at https://github.com/JoseBarbero/CamPype.

**Supplementary Information:**

The online version contains supplementary material available at 10.1186/s12859-023-05414-w.

## Background

Since the Human Genome Project was completed in 2003 [1], Whole-Genome Sequencing (WGS) costs are substantially decreasing over time, which has led to the emergence of new sequencing technologies that empower Next-Generation Sequencing (NGS) based on Sequencing by Synthesis (SBS), Sequencing by Oligo Ligation Detection (SOLiD), Single-Molecule Real-Time (SMRT) sequencing and nanopore-based DNA sequencing [2, 3]. Among them, Illumina sequencing remains one of the most prevalent sequencing technologies providing high accuracy and coverage with low error rates (< 1%), compared to Pacific Biosciences or Oxford Nanopore technologies, that can afford much longer read lengths but with higher error rates and lower accuracy [4, 5].

The development of WGS has revolutionized microbiology research practices by replacing many traditional time-consuming and labor-intensive techniques [6]. Genome sequences can be obtained in a matter of hours, compared to the days or weeks required for the conventional laboratory methods and tests for completion, including Pulse-Field Gel Electrophoresis (PFGE), serotyping and phenotypic tests [7, 8]. In clinical microbiology, patient diagnosis time has been significantly reduced providing wider diagnostics repertoire. Current applications of WGS in this field include clinical identification from primary samples, infection control actions, antimicrobial stewardship, outbreak detection and intervention and pathogen discovery [9]. In food safety, EFSA (European Food Safety Authority) and FDA (Food and Drug Administration) are applying WGS of foodborne pathogens for microbial risk assessments and regulatory purposes [10, 11]. Even more, WGS is being widely used to study the microbial ecology of food products and environments along the food supply chain [12].

The implementation of WGS in clinical and food microbiology laboratories has led to the establishment of large public databases comprising thousands of genomes available [13]. The vast amounts of data produced by NGS require advanced bioinformatics skills for efficient WGS analysis, which normally are not acquired by researchers. This is one of the main bottlenecks for every microbiology laboratory in the application of WGS as a routine laboratory technique [14]. To overcome this obstacle, bioinformatics workflows are constantly being developed for the automatic analysis of genome sequences and many of them are designed for researchers without bioinformatics expertise, such as TORMES [15], BacPipe [16], ASA^3^P [17] and Bactopia [18]. However, none is specially intended in the genera *Campylobacter*, that is the main cause of gastroenteritis worldwide [19]. Most campylobacteriosis cases (> 90%) are caused by *Campylobacter jejuni,* while *Campylobacter coli* is responsible for almost 10%. These bacteria are ubiquitous and live in the intestinal tract of poultry, pigs and cattle, but they may also be found in the feces [20]. Their genome is relatively small with ~ 1.6–1.8 Mbp length and a G + C content around 30–32% and encodes a rich inventory of virulence genes and antibiotic resistance markers responsible for their pathogenicity [21]. Moreover, campylobacteriosis is estimated to cost the EU public health systems around 2.4 billion euros per year [22]. Thus, an automated workflow for *Campylobacter* spp. would accelerate epidemiological studies through the different sequencing-based typing methods that have arisen since the first *Campylobacter* genome was published in 2000 [23], such as ribosomal Multilocus Sequence Typing (rMLST), core-genome MLST (cgMLST) or whole-genome MLST (wgMLST), that ultimately would help to reduce the costs of campylobacteriosis outbreaks.

In this work, we present CamPype, an open-source workflow for the WGS analysis of paired-end Illumina reads from *C. jejuni* and *C. coli*, although any other bacterial genus can be analyzed as well. CamPype includes a fully customizable configuration, leading to the specific results the researchers want and saving time running steps they do not need. The entire workflow can be run using one single command, making it easy to use for researchers that are not familiar with the command line. Also, CamPype provides conda environments (https://docs.anaconda.com/) with Bioconda packages [24] for all the dependencies it needs, avoiding incompatibilities between them and making the installation as easy as possible. Finally, CamPype integrates a graphical HTML report that includes the results of every tool in the workflow shown in a more illustrative manner, allowing the researchers to access the results of the analysis easily and at a simple glance.

## Implementation

### CamPype analysis workflow

**Fig. 1:**
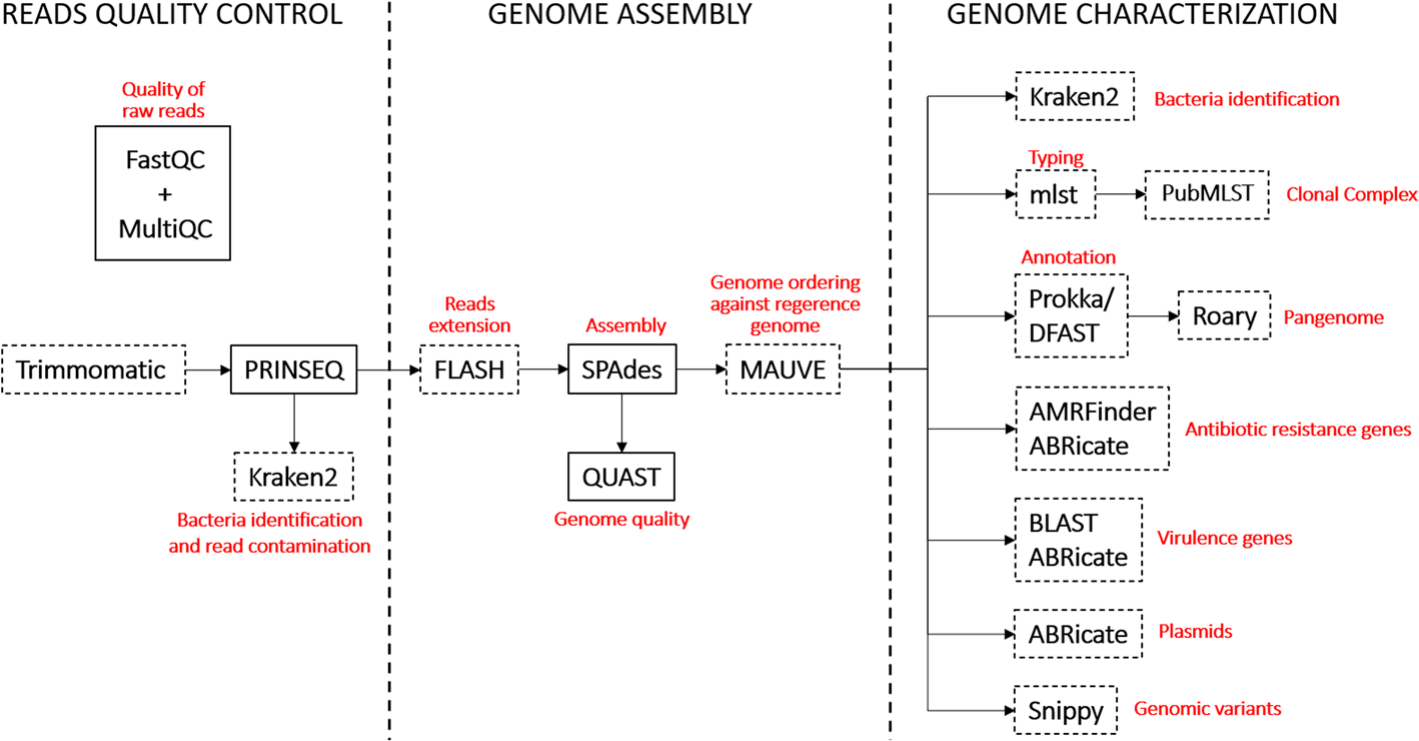
Summary of the CamPype analysis workflow. Evaluation of sequencing raw data can be performed independently and previously to CamPype. Bacteria identification is performed on the filtered fastq reads when raw reads are provided or after genome assembly when contigs are used. Software or databases are indicated in boxes. Discontinuous boxes indicate tools that users can deactivate

The CamPype workflow comprises three main stages (read quality control, genome assembly and genome characterization) that include several processes conducted by different tools. CamPype can take raw reads or assembled genomes in contigs as inputs. Instructions to set up the input files and workflow configuration are addressed in the CamPype repository (https://github.com/JoseBarbero/CamPype). Users can skip certain processes and adjust the configuration of parameters and databases from among the different options included for each stage in the *campype_config.py* file. An overview of the structure of CamPype is summarized in Fig. 1.

#### Read quality control

Previously to CamPype, a sequencing data analysis can be performed with FastQC (S. Andrews, http://www.bioinformatics.babraham.ac.uk/projects/fastqc) to assess the quality of raw reads and optimize the step of read quality control. For fast visualization of this analysis, MultiQC [25] combines these results into a single interactive HTML. Then, sequencing adaptors can be trimmed from raw sequencing reads through Trimmomatic [26] by using the *trim_adaptors* option. CamPype includes all possible Illumina adaptor sequences in the file indicated in the option *adapters_reference_file*, although users can include their own. Then, reads are quality filtered and trimmed by PRINSEQ [27] according to specific parameters. These are the minimum read length (*min_len*), minimum read quality (*min_qual_mean*), quality threshold score from the 3'-end to trim sequence by (*trim_qual_right*) and sliding window size (*trim_qual_window*). The reads that pass the quality control can then be used for bacterial identification and read contamination by Kraken2 [28] using the *species_identification* option*.*

#### Genome assembly

The reads that pass the quality control can be extended using FLASH [29] (*merge_reads*) and merged and unmerged reads are further de novo assembled using SPAdes [30]. The assembly mode and k-mer size(s) to be used can be selected using the *mode* and* k* options, respectively. Quality assembly of genomes is evaluated with QUAST [31] and contigs below the minimum length provided by *min_contig_len* are discarded. Resulted contigs are ordered using progressiveMauve [32] against a reference genome when specified in the options included under the *reference_genome* block.

#### Genome characterization

Draft genomes (ordered or not) can be further characterized through different tools, which can be selected or disabled by using the corresponding options. These include software for taxonomic classification, Multi-Locus Sequence Typing (MLST), genome annotation, detection of antibiotic resistance genes, virulence genes and plasmids, pangenome construction and identification of SNPs. For bacterial identification, Kraken2 [28] assigns taxonomic labels to draft genomes (*species_identification*) when assembled genomes are used as inputs. For subtyping purposes, MLST is performed through mlst (T. Seemann, https://github.com/tseemann/mlst) using the *run_mlst* option, and Clonal Complexes (CCs) are assigned with the *Campylobacter jejuni*/*coli* PubMLST scheme [33] using the *include_cc* option. Prokka [34] or DFAST [35] can be used to annotate genomes by using the *annotator* option, although this stage can be disabled by using the *run_annotation* option. A reference genome annotation in GenBank format to first annotate from can be used in Prokka [34] through the *reference_annotation* option. Keeping the raw product annotation (*rawproduct*) in Prokka [34] is highly encouraged to reduce number of hypothetical proteins. DFAST [35] includes pseudo/frameshifted gene prediction and conserved domain search. The “gff” files generated are used by Roary [36] to construct the pangenome based on the presence/absence of predicted genes. Pangenome summary figures are created using the *roary_plots.py* script by Marco Galardini (https://github.com/sanger-pathogens/Roary/blob/master/contrib/roary_plots/roary_plots.py) with minor modifications to show isolate labels and CCs (when possible) in the presence/absence accessory genome tree. Paralogs split can be disabled by the option *split_paralogs* and minimum percentage of identity for blastp can be selected by using the *minid* option. Moreover, pangenome analysis can be skipped by using the *run_pangenome* tool. Antibiotic resistance genes can be searched using protein alignments with AMRFinderPlus [37] against the NCBI Bacterial Antimicrobial Resistance Reference Gene Database (BioProject PRJNA313047), that will also identify resistance-associated point mutations only for *Campylobacter* spp., or/and using nucleotide alignments with ABRicate (T. Seemann, https://github.com/tseemann/abricate) against any of the databases provided by this tool (*antimicrobial_resistance_databases*), such as ARG-ANNOT [38], CARD [39], MEGARes [40], the NCBI Bacterial Antimicrobial Resistance Reference Gene Database (BioProject PRJNA313047) or/and ResFinder [41]. Antibiotic resistance genes searching can be skipped using the *run_antimicrobial_resistance_genes_prediction* option and specific tools can be selected in the *antimicrobial_resistance_genes_predictor_tool* option. Draft genomes can be also screened for virulence genes using tBLASTn against an in-house database (*proteins_reference_file*), or/and BLASTn with ABRicate (T. Seemann, https://github.com/tseemann/abricate) against any of the databases provided by this tool (*virulence_factors_databases*), such as the Virulence Factors Database (VFDB) [42]. Users are encouraged to increase the size of the in-house *Campylobacter* spp. database provided with more sequences of interest or create a new one for other species, while checking the databases available in ABRicate at its repository (T. Seemann, https://github.com/tseemann/abricate). Activation of *soft_masking* is highly encouraged to find initial matches when using tBLASTn. Virulence genes search can be skipped using the *run_virulence_genes_prediction* option and specific tools can be selected in the *virulence_genes_predictor_tool* option. Minimum identity (*minid*) and coverage (*mincov*) can be selected within each tool for considering an antibiotic resistance gen and virulence gen as present. Plasmids are searched using BLASTn and ABRicate (T. Seemann, https://github.com/tseemann/abricate) against the PlasmidFinder database [43], although the analysis can be disabled by using the *run_plasmid_prediction* option. Genetic variants identification is performed through snippy (T. Seemann, https://github.com/tseemann/snippy) using the reference genome indicated in the *file* option below the *reference genome* options, as mentioned before.

Last, a summary HTML report is generated to resume the results of CamPype and can be displayed on any web browser. The report is generated in R environment (https://www.R-project.org/) using the following R packages: ape [44], complexheatmap [45], dplyr (https://CRAN.R-project.org/package=dplyr), DT (https://CRAN.R-project.org/package=DT), ggplot2 [46], ggtree [47], pander (https://CRAN.R-project.org/package=pander), plotly [48], rjson (https://CRAN.R-project.org/package=rjson), rmarkdown (https://rmarkdown.rstudio.com) and tidyverse [49]. The report includes data summary, interactive tables and figures that can be copied or downloaded. An example of an analysis report can be found at https://josebarbero.github.io/CamPype/example_report/CamPype_Report_long_first_case_study.html.

The results of CamPype are stored in specific directories for each stage and tool, with separate folders for each isolate, and include log files for analysis tracking and results standardization across different users together with files that compare the results across all analyzed samples. The location and name of the CamPype output directory can be set using the options *output_directory* and *custom_output_name*, although by default date and time of execution will be added to the directory name for managing analyses. Moreover, CamPype allows the use of multiple threads to accelerate the analysis (*n_threads*).

### Hardware and software setup

The CamPype workflow was developed using a combination of python v3.7.8, GNU bash v5.0.17 (https://www.gnu.org/software/bash/) and R v4.1.3. CamPype is freely available at https://github.com/JoseBarbero/CamPype with a detailed instruction manual for its installation and use on any UNIX operating system. The CamPype workflow, including all required tools and dependencies, can be automatically installed using the conda environment provided. The execution of CamPype requires enough storage space. It is recommended to have available at least three times the size of the input data for a successful complete execution when raw reads are taken as inputs and one or two GB of free space in hard disk when contigs are taken as inputs (although the genomic variant calling also requires ~ 250 MB per genome). For the study case reported here, we used a high computational capability (28 CPU cores and 64 GB RAM), even though CamPype can be run in any standard computer.

### Validation of CamPype’s functionality: two case studies

#### Analysis of *Campylobacter jejuni* and *Campylobacter coli* strains (input: raw reads)

Ten previously published and WGS analyzed (raw reads) *C. jejuni* (5) and *C. coli* (5) strains isolated from faeces of *Bos taurus* and *Ovis aries* [50] were used to test CamPype workflow. DNA was extracted using the NZY Microbial gDNA Isolation kit (NZYtech) and sequenced using Illumina NovaSeq6000 with the NEBNext Ultra™ II FS DNA Library Prep Kit (Illumina, San Diego, CA, USA). Raw reads deposited in the European Nucleotide Archive (ENA) at EMBL-EBI under accession numbers SAMN17214749 (strain C0430), SAMN17214753 (strain C0455), SAMN17214754 (strain C0459), SAMN17214757 (strain C0538), SAMN17214765 (strain C0551), SAMN17214771 (strain C0561), SAMN17214781 (strain C0582), SAMN17214797 (strain C0642), SAMN17214804 (strain C0663) and SAMN17214806 strain (strain C0669), were directly analyzed using CamPype with default configuration.

#### Analysis of *Escherichia coli* genomes (input: contigs)

A total of 44 assembled genomes of *Escherichia coli* randomly selected from the RefSeq database were used as input for CamPype: GCF_003017915.1 (strain 2014C-3051), GCF_003018035.1 (strain 2015C-4944), GCF_003018055.1 (strain 2013C-3252), GCF_003018135.1 (strain 2014C-3050), GCF_003018315.1 (strain 2013C-3513), GCF_003018455.1 (strain 97–3250), GCF_003018555.1 (strain 2013C-4225), GCF_003018575.1 (strain 2013C-4538), GCF_003018795.1 (strain 2012C-4606), GCF_003018895.1 (strain 2014C-3057), GCF_003019175.1 (strain 2013C-4187), GCF_004010675.1 (strain 2010C-3347), GCF_004010715.1 (strain 08–3914), GCF_025995195.1 (strain F690), GCF_025995255.1 (strain F765), GCF_025995315.1 (strain H52_982342), GCF_025995355.1 (strain 8_140198), GCF_025995415.1 (strain 26_141088), GCF_025995475.1 (strain 27_141091), GCF_025995535.1 (strain 53_142304), GCF_025995615.1 (strain 57_142493), GCF_025995675.1 (strain 61_150228), GCF_025995735.1 (strain 93_161312), GCF_025995895.1 (strain CEC96047), GCF_025996315.1 (strain CEC13091), GCF_025996495.1 (strain CEC08123), GCF_025996555.1 (strain CEC03102), GCF_025996675.1 (strain CEC13002), GCF_025996735.1 (strain CEC13004), GCF_027925505.1 (strain 2313), GCF_027925565.1 (strain EH031), GCF_027925625.1 (strain H19), GCF_027925685.1 (strain 20–1), GCF_027925745.1 (strain EH2252), GCF_027925765.1 (strain 98E11), GCF_027925785.1 (strain NIID080884), GCF_027925805.1 (strain PV0838), GCF_027925825.1 (strain 10,153), GCF_027925845.1 (strain 02E060), GCF_008926165.1 (strain ERL06-2442), GCF_005221885.1 (strain 143), GCF_008931135.1 (strain ERL04-3476), GCF_005221505.1 (strain 150) and GCF_008926185.1 (strain ERL05-1306). The default configuration of CamPype was modified as follows. The genome and annotation of *Escherichia coli* strain K-12 from NCBI (NZ_CP047127) were used as reference (*reference_genome*), the *assembled_genomes* option was set to True, the *include_cc* option was set to False, ABRicate was used for virulence genes screening (*virulence_genes_predictor_tool*), and variant calling was set to True (*run_variant_calling*).

## Results

**Fig. 2:**
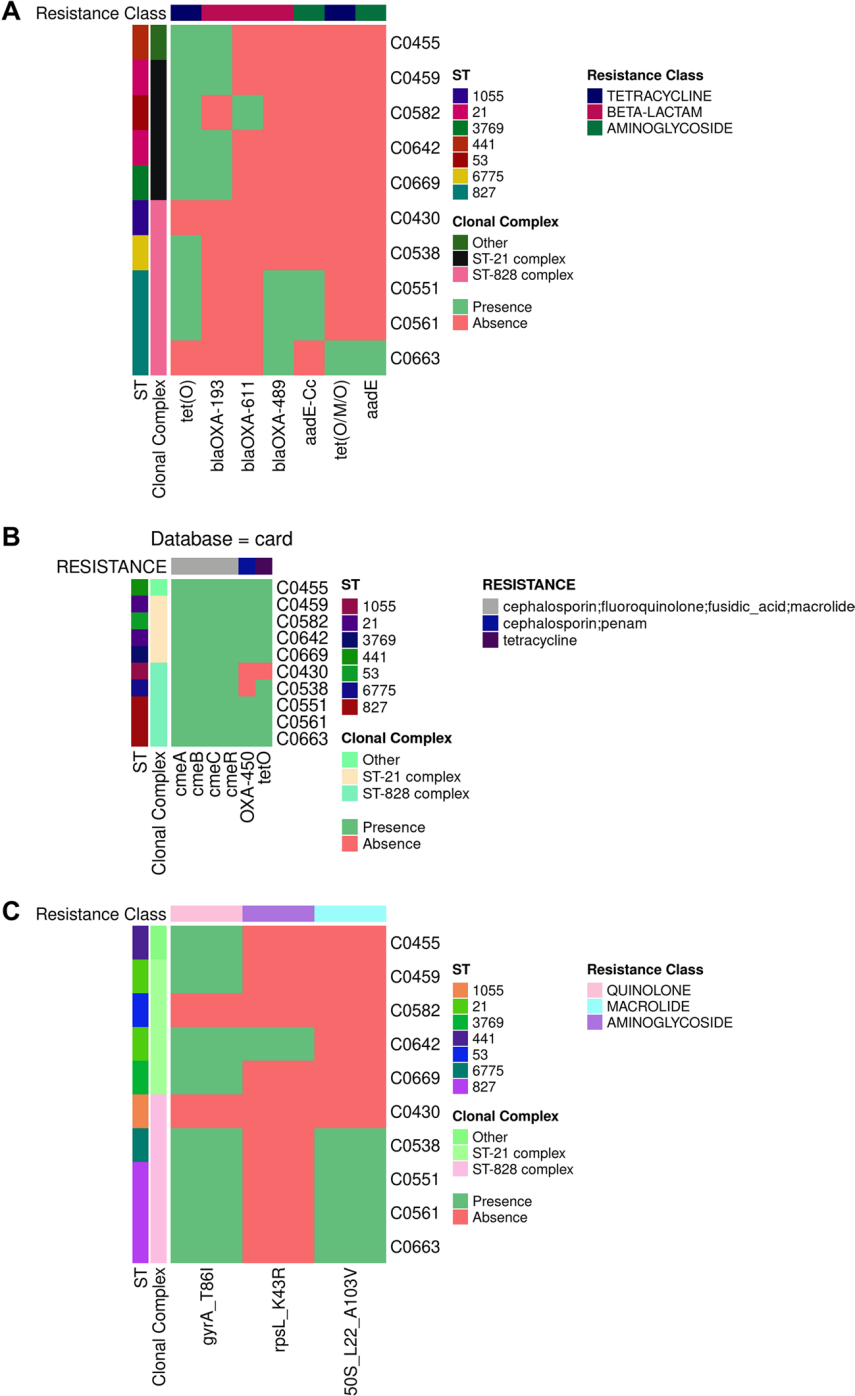
Antibiotic resistance markers identified in the *Campylobacter jejuni* and *Campylobacter coli* isolates included in the first case study. Prevalence of antibiotic resistance genes were determined with the NCBI database and AMRFinder **A**, and the CARD database and ABRicate **B**. Point mutations conferring antibiotic resistance **C** were determined with the NCBI database and AMRFinder

**Fig. 3:**
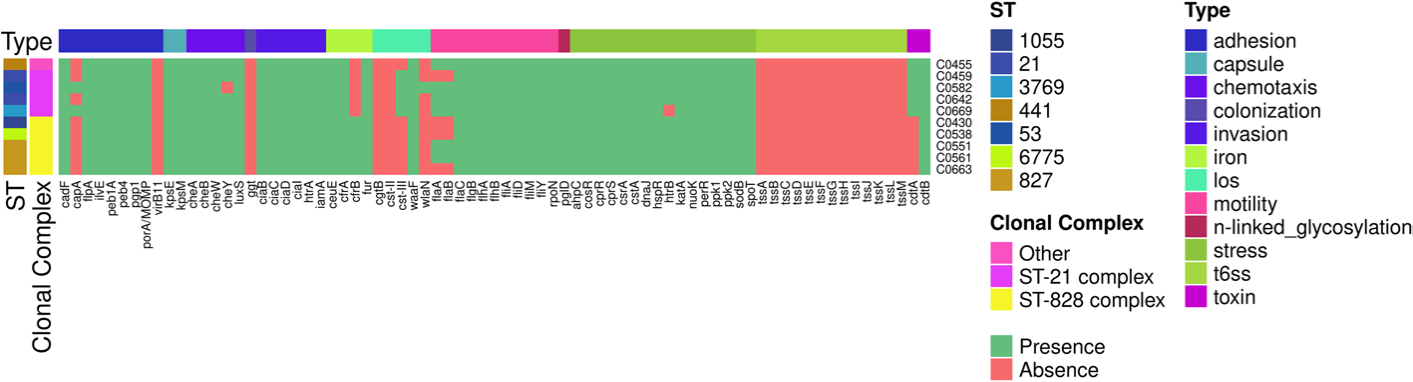
Virulence genes identified in the *Campylobacter jejuni* and *Campylobacter coli* isolates included in the first case study. Prevalence of the 76 genes comprising the inhouse database of virulence factors was evaluated with tBLASTn. For each isolate, the Sequence Type (ST) and Clonal Complex (CC) is indicated. For each gene, the virulence factor category is indicated

**Fig. 4:**
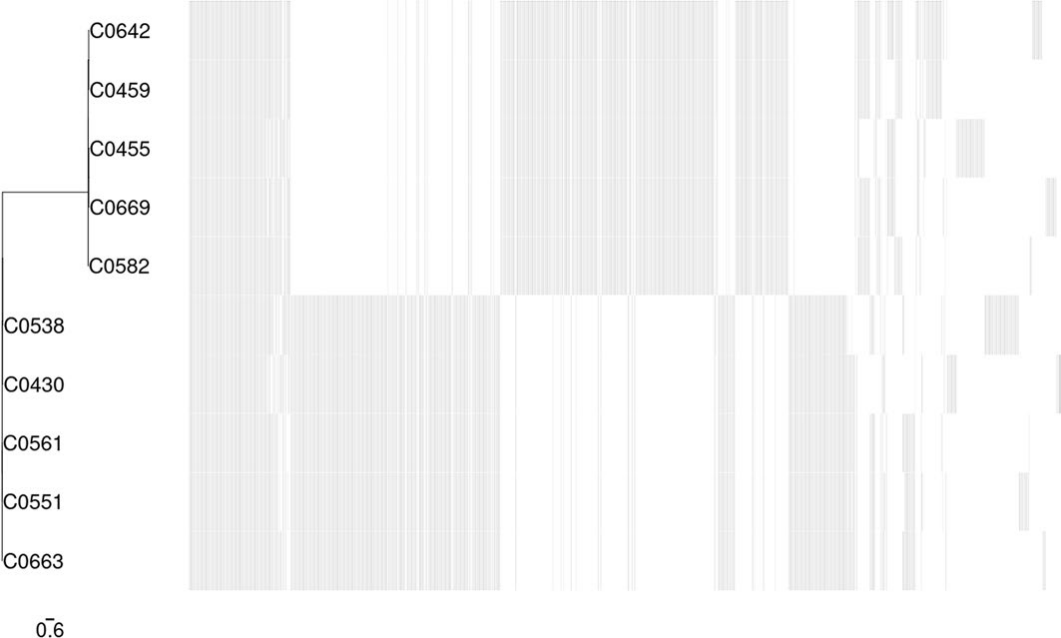
Gene presence/absence analysis of the *Campylobacter jejuni* and *Campylobacter coli* isolates included in the first case study. Roary was used to create the pangenome and the binary presence/absence of accessory genes was used to construct the tree. Genes (columns) coloured in grey are present in each isolate (rows), whereas genes coloured in white are absent in each isolate

Here, the analysis of ten *C. jejuni* and *C. coli* isolate sequences is reported to validate CamPype workflow. The analysis took 5.4 h using 28 CPUs and generated a result directory of 17.2 GB (from 9 GB of compressed input data). The results of the raw reads quality control can be found in https://josebarbero.github.io/CamPype/example_report/multiqc_report_first_case_study.html, and the report with the summarized results generated by CamPype can be visualized in https://josebarbero.github.io/CamPype/example_report/CamPype_Report_long_first_case_study.html. A total of 13.0 M ± 2.5 M reads per sample were directly submitted to CamPype and reduced to 12.2 M ± 2.3 M reads per sample by the quality control stage; i.e., 97% of reads survived overall and 30% were then merged (Additional file 1). The assembly yielded 1.6–1.7 Mbp-long draft genomes fragmented into 10 to 41 contigs, corresponding to an average coverage of 1102 ± 207X with mean N50 of 242 ± 103 kbp and overall GC content of 30.4% in *C. jejuni* and 31.4% in *C. coli*. MLST revealed that isolates belonged to 7 defined Sequence Types (STs) (ST-21, ST-53, ST-441, ST-827, ST-1055, ST-3769 and ST-6775) that were grouped into Clonal Complexes (CCs) CC21 (*C. jejuni*) and CC828 (*C. coli*). Most isolates (100% *C. jejuni* and 60% *C. coli*) harbored a *bla*_*OXA*_ gen and *tet(O)*, conferring resistance to beta-lactams and tetracyclines, respectively. *C. jejuni* strains harbored *bla*_*OXA-193*_ or *bla*_*OXA-611*_, whereas *C. coli* strains C0551, C0561 and C0663 harbored *bla*_*OXA-489*_ (Fig. 2A). No antibiotic resistance gen was found in *C. coli* strain C0430, while *C. coli* strain C0538 presented only the *tet(O)* gen. Moreover, resistance to aminoglycosides (*aadE* or *aadE-Cc*) was only found in *C. coli* (60%). The efflux systems CmeABC and CmeDEF and the *CmeR* repressor were present in all isolates (Fig. 2B). The point mutation *gyrA* p.T86I conferring resistance to quinolones was present in 80% of both species, and the point mutation *rpsL* p.K43R conferring resistance to streptomycin was only found in *C. jejuni* strain C0642, while the 50S rRNA L22-A103V point mutation was only found in *C. coli* (80%) (Fig. 2C). TBLASTn against an in-house database of 76 sequences was used for virulence genes searching and 52 to 57 virulence genes were found among all isolates (Fig. 3). Differences among the ten isolates were found for the following ten genes: *capA, cdtA, cdtC, cfrB, cheY, cst-III, flaA, flaB, htrB* and *wlaN*. The genes *virB11*, *ggt*, *cgtB*, *cst-II* and the 13 genes of the Type VI Secretion System (T6SS) were not found in any of the isolates. No plasmids were found in any of the isolates. A total of 1657–1806 Coding DNA Sequences (CDS) were annotated among all isolates (Additional file 1) and were further grouped in 3575 gen clusters in the pangenome, of which 314 genes (9%) were present in all isolates (Fig. 4).

CamPype reproduced the results included in the publication of Ocejo et al. [50], that were MLST, antibiotic resistance determinants and plasmids screening in the ten assembled *Campylobacter* spp. genomes in 31 to 63 contigs of such publication.

Besides, the analysis of the 44 genomes of *E. coli* took 5.5 h and produced a directory of 13.6 GB (from 74.7 MB of compressed input data), of which 10.4 GB constituted the genomic variants calling directory. The CamPype’s HTML report for the analysis of the *E. coli* genomes can be found in https://josebarbero.github.io/CamPype/example_report/CamPype_Report_short_second_case_study.

## Discussion

Advances in NGS has transformed the fields of clinical and food microbiology [51, 52]. The impact is such that WGS is now routinely applied as the reference standard for infection control and epidemiology and pathogen typing [53]. WGS allows the most detailed characterization possible of bacteria to date by enabling a resolution unattainable compared to conventional laboratory typing methods with much higher level of certainty [54]. However, the large data sets generated from sequencing technologies require advanced bioinformatics training to properly use the tools and interpret the results obtained [55]. Even so, automated workflows are a rapid solution for microbiologists to allow fast and efficient analysis of data [56]. Here, the robustness of CamPype to handle *Campylobacter* WGS reads obtained from Illumina paired-end sequencing technologies is demonstrated through two different scenarios. Ten previously published *C. jejuni* and *C. coli* genomes were analyzed from the sequencing raw data using CamPype in a single command and produced same results to that of the multi-stage analyses included in the publication of Ocejo et al. [50], and even with reduced number of assembled contigs. Additional data not reported in the aforementioned study was also generated through CamPype to complement the WGS analysis, including extended statistics of reads, assembly and annotation, detection of virulence genes, and pangenome construction, all of which were showed in an attractive HTML report. In addition, CamPype behaved efficiently for assembled genomes of different species, demonstrating the successful performance of this workflow for processing varying amounts of genomic sequencing data from diverse origin. For that second scenario, the default configuration was properly adjusted to analyze 44 previously published *E. coli* genomes using contigs as input and results were accurately reported for each genome, including bacterial typing (MLST), assembly analysis and genome annotation, searching for antibiotic resistance genes, virulence genes and plasmids, pangenome construction and identification of nucleotide variants against *E. coli* str. K-12 as reference genome. The most outstanding and promising tools hitherto for WGS are available for the users to include in the analysis, and their parameters can also be adjusted to meet their preferences. CamPype integrates various alternatives to identify antibiotic resistance genes and virulence genes since there is no single standardized and open-access database for antimicrobial resistance targets or virulence factors identification, so that the supplementary use of sequence databases generates the most complete results possible [57]. The combination of diverse data sources with different records is an excellent strategy to get partial but complementary information [58]. This is the starting point toward advancing in precision medicine for effective target therapies, as more information becomes available through the use of WGS approaches [59]. Moreover, certain analysis can be skipped to generate results in shorter times providing faster turnaround times, which has the advantage of favoring therapeutic decision-making as well. Additionally, the output of CamPype can easily be used to study epidemiological outbreaks through phylogenetic analyses of genomic variants. CamPype can handle either raw reads or assembled contigs, giving great flexibility for users and broadening its application not only for clinical diagnostic and food safety laboratories, but also towards epidemiology and comparative genomics studies.

CamPype is specially developed for *C. jejuni* and *C. coli* as they are the main responsible of gastroenteritis in humans with a frequency of about 3–4 times higher than in *Salmonella* or *E. coli* [60]. The possibility of grouping *C. jejuni* and *C. coli* Sequence Types into Clonal Complexes while providing a specific virulence genes database of this genus were not found in any of the existing microbial analysis pipelines to date, such as TORMES [15], BacPipe [16], ASA^3^P [17] and Bactopia [18]. Moreover, opposite to these currently available pipelines, CamPype offers the option to evaluate the quality of sequencing data for optimal read quality filtering. Nonetheless, isolates from any genera and origin can be analyzed as well using CamPype. The routine use of WGS as a primary prevention is an economic favorable priority for the control of foodborne infection and other serious hospital-associated infections [61]. A mathematical simulation modelling study highlighted the direct hospital cost savings and outbreaks sizes reduction of using WGS compared to standard medical care practices [62]. The web-like report generated in CamPype provides a quick insight into antibiotic resistance targets and virulence genes facilitating a faster and accurate response in time-critical situations with lower healthcare costs [53]. Thus, CamPype would definitely help in *Campylobacter* infection control actions to minimize adverse patient outcome and in outbreak investigation. Besides, the workflow has been already used for the characterization of *Campylobacter jejuni*-associated with perimyocarditis [63] and also for comparative genomics analysis of hundreds of *Campylobacter* spp. isolated from Spain (*in prep.*).

CamPype was developed with the needs of microbiology laboratories in mind and obstacles that restrict the use of WGS for clinical/public health microbiology investigations [56]. Along with being user-friendly and customizable, CamPype is a comprehensive workflow that is capable of performing a very detailed automated analysis of large numbers of genomes in a single process without previous specific knowledge and bioinformatics skills, by using simple commands. The open-source nature allows collaborative coding between users and developers with the intention to fulfill users' needs and be improved through as many suggestions as proposed by the community to make CamPype an outstanding workflow. The analysis is performed locally, which means the user is the owner of the data in every moment without needing internet connection. Other free resources, such as Galaxy [64] and PATRIC [65], integrate attractive and interactive user interfaces, but require a fast and consistent internet connection for importing data to the server that can lead to privacy and security issues with data protection policies varying between countries [66]. Moreover, the performance of analysis depends not only on the number of raw reads but also on the hardware of the computer used, with reduced execution time when more CPU processors are available, whereas web-served based analyses take indeterminate execution times that vary on the server workloads, which is unreliable for patient care emergency situations [67].

## Conclusions

Implementing WGS in clinical and food microbiology laboratories has led to an increase in the amount of raw data and genomes publicly available. However, the use of WGS as a routine method is unfeasible without the application of bioinformatics resources and remains a challenge due to the required specific skill set. CamPype is a reliable solution for integration WGS into routinely use and overcome these barriers because it enables easy and automated analysis of large genome datasets, providing a quick visualization of results that facilitates data interpretation.

## Supplementary Information

**Additional file 1: **Genomic characteristics of the Campylobacter jejuni and Campylobacter coli isolates included in the first case study.

## Data Availability

The datasets supporting the conclusions of this article are included within the article (and its additional file). The CamPype software is available at https://github.com/JoseBarbero/CamPype and an example of the analysis report is provided in https://josebarbero.github.io/CamPype/example_report/CamPype_Report_long_first_case_study.html. The raw reads and assembled genomes used to test CamPype can be found in https://zenodo.org/record/7999130.

## Abbreviations

CC: Clonal complex
CDS: Coding DNA sequence
cgMLST: Core-genome MLST
EFSA: European Food Safety Authority
ENA: European Nucleotide Archive
FDA: Food and Drug Administration
GC: Guanine-cytosine
MLST: Multilocus sequence typing
NGS: Next-generation sequencing
PFGE: Pulse-field gel electrophoresis
rMLST: Ribosomal MLST
SBS: Sequencing by synthesis
SMRT: Single-molecule real-time
SNP: Single-nucleotide polymorphism
SOLiD: Sequencing by oligo ligation detection
ST: Sequence type
T6SS: Type VI secretion system
VFDB: Virulence factors database
wgMLST: Whole-genome MLST
WGS: Whole-genome sequencing
